# Therapeutic Monoclonal Antibodies as Advanced Therapies for Companion Animals: Species Adaptation, Fc Biology, Clinical Translation, and Future Platforms

**DOI:** 10.3390/vetsci13080778

**Published:** 2026-08-03

**Authors:** Ying Fu, Zhiling Guo, Huiyan Wang

**Affiliations:** Jilin Collaborative Innovation Center for Antibody Engineering, Jilin Medical University, Jilin 132013, China; fuying@jlmu.edu.cn (Y.F.); 13844673829@163.com (Z.G.)

**Keywords:** companion animals, monoclonal antibody, caninisation, felinisation, Fc biology

## Abstract

Monoclonal antibodies are emerging as advanced therapies in companion-animal medicine, with approved or clinically evaluated applications in canine and feline osteoarthritis pain, canine atopic dermatitis, and selected infectious-disease and oncological settings. This critical narrative review integrates peer-reviewed studies, regulatory records, product information, and publicly available therapeutic-antibody sequence resources to examine target biology, species adaptation, Fc function, developability, and clinical implementation. Anti-NGF and anti-IL-31 products demonstrate the clinical value of long dosing intervals, whereas oncology and infectious-disease programmes remain subject to indication-specific efficacy and safety requirements. Canine and feline IgG subclasses, Fc-receptor and FcRn biology, incomplete feline framework resources, manufacturing economics, and owner-funded care all influence candidate design and deployment. Emerging bispecific antibodies, antibody–drug conjugates, VHHs, mRNA-based approaches, and AI-assisted strategies are most relevant when they address a defined veterinary use case. We propose a translational framework linking target validation, species-adapted engineering, species-matched assays, manufacturability, regulatory evidence, and real-world deployment.

## Simple Summary

Monoclonal antibodies are becoming increasingly practical advanced therapies for dogs and cats. Products that neutralise nerve growth factor can reduce osteoarthritis-associated pain, and an antibody targeting interleukin-31 can control pruritus in dogs. These successes show that antibody therapy can improve mobility, comfort, and quality of life while enabling dosing intervals compatible with routine veterinary visits. However, a human antibody programme cannot simply be transferred to another species. The targeted pathway must be biologically relevant in the intended animal population, and the antibody must be adapted to canine or feline immune biology, manufacturing requirements, veterinary-clinic workflows, safety monitoring, and owner-funded care. This review explains how target selection, caninisation or felinisation, Fc function, pharmacokinetics, clinical trial design, and regulatory evidence jointly determine whether an antibody can become a useful veterinary medicine. It also evaluates emerging bispecific antibodies, antibody-drug conjugates, single-domain antibodies, mRNA-based delivery, and AI-assisted design. The central message is that technological complexity is valuable only when it addresses a defined clinical problem and improves the health, welfare, or quality of life of companion animals.

## 1. Introduction

Approved products have established that monoclonal antibodies can provide clinically useful treatment in dogs and cats. Their target specificity can be combined with dosing intervals that align with routine veterinary visits, which is particularly valuable in chronic disease. In canine pain management, for example, antibody therapy may reduce reliance on repeated NSAID administration, for which gastrointestinal and renal toxicity remain important constraints [1,2]. Bedinvetmab and frunevetmab illustrate how this clinical need has been translated into veterinary programmes for osteoarthritis pain [3,4].

Development nevertheless occurs within a much smaller and less complete evidence base than that available for human therapeutics. Dogs and cats differ from humans in IgG-subclass architecture, Fc-receptor biology, and neonatal Fc receptor (FcRn) kinetics. Their germline repertoires are also less completely characterised for CDR grafting, particularly in cats. In addition, veterinary approval pathways apply marketing-authorisation and data requirements that differ from those used for human medicines [5,6]. These factors shape framework selection, Fc design, assay development, manufacturing strategy, and the interpretation of clinical evidence.

Previous reviews have largely focused on individual products and their clinical use. This review instead treats the companion-animal mAb entries in Thera-SAbDab as an engineering cohort and examines how species adaptation, Fc biology, public framework resources, and CMC constraints shape development decisions. The discussion moves from approved and clinical-stage products to IgG-subclass biology and species adaptation before examining emerging targets, next-generation formats, and a research agenda for companion-animal antibody engineering [7].

### Review Scope, Evidence Sources, and Translational Framework

This critical narrative review examines the development of therapeutic monoclonal antibodies for dogs and cats across the continuum from target validation and species adaptation to Fc selection, developability, clinical evaluation, and deployment in veterinary practice. A structured literature search was conducted in PubMed, Web of Science Core Collection, and Scopus from database inception to 15 July 2026 using combinations of the terms “companion animal”, “dog”, “canine”, “cat”, “feline”, “monoclonal antibody”, “therapeutic antibody”, “caninization”, “felinization”, “Fc receptor”, “FcRn”, “antibody engineering”, and “veterinary immunotherapy”. Reference lists of relevant articles were also examined to identify additional primary studies. Thera-SAbDab was used to identify publicly available therapeutic-antibody sequence records, whereas regulatory status, approved indications, and product information were independently verified using records from the European Medicines Agency, the U.S. Food and Drug Administration Center for Veterinary Medicine, and the U.S. Department of Agriculture Center for Veterinary Biologics [7]. Database inclusion was therefore treated as evidence of public sequence availability rather than as proof of regulatory approval, clinical efficacy, or current commercial status.

Evidence was interpreted according to its relevance to the claim being assessed. Regulatory assessment reports and peer-reviewed clinical studies were prioritised for product status, indication, efficacy, safety, dosing, and pharmacokinetics. Primary species-specific studies were prioritised for target biology, IgG-subclass function, Fc-receptor interactions, FcRn biology, and variable-region adaptation. Manufacturer communications, patent documents, conference materials, and other programme records were considered only when regulatory or peer-reviewed evidence was unavailable, and their evidentiary limitations were explicitly acknowledged. Recent developments were included to reflect the rapidly evolving therapeutic landscape, including the 2025 authorisation of the anti-NGF antibodies relfovetmab and izenivetmab, the first pilot clinical evaluation of a caninised anti-CTLA-4 antibody in dogs, and the 2026 multicentre clinical evaluation of gilvetmab [8,9,10,11].

**Figure 1:**
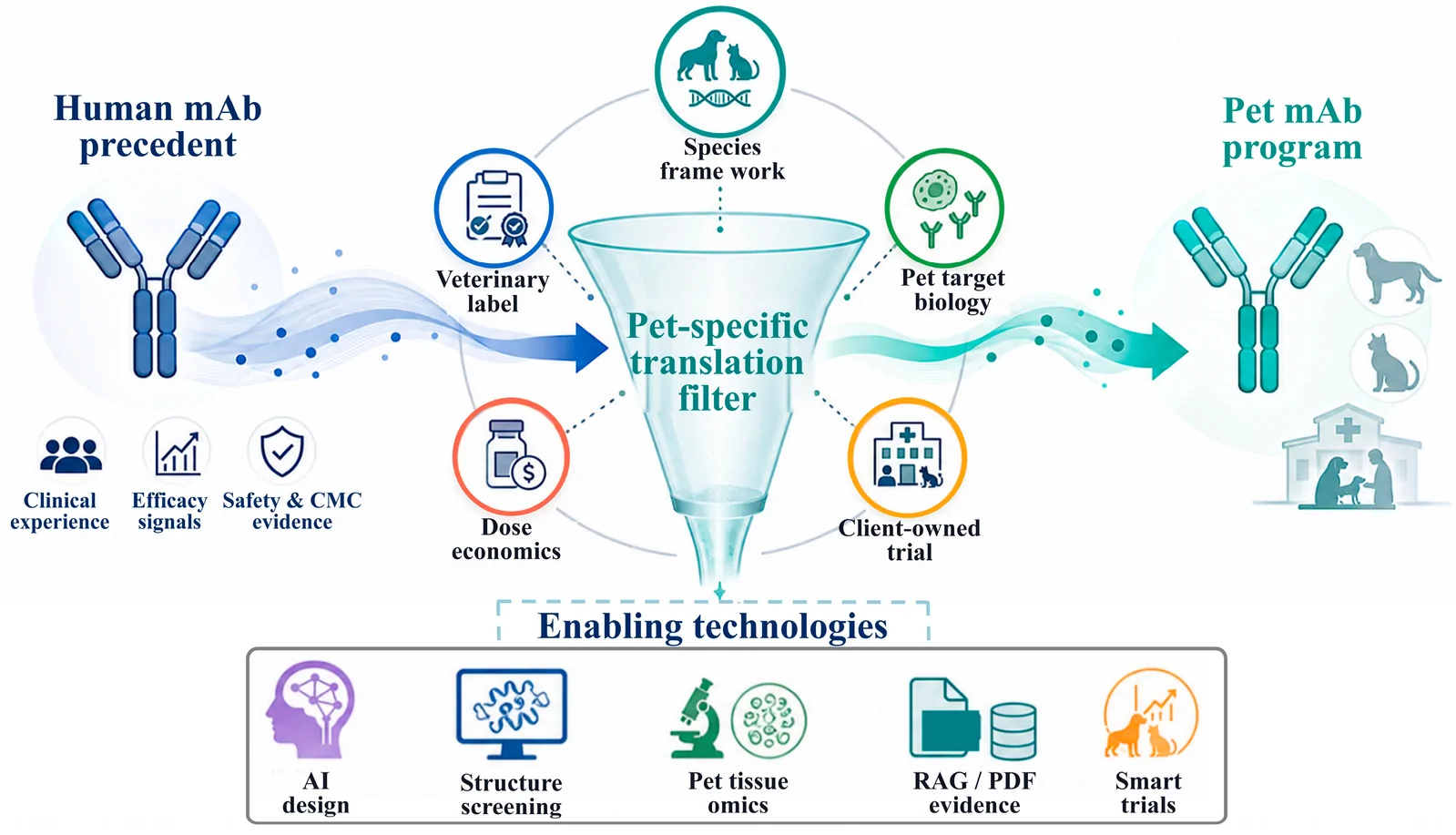
Translational framework for companion-animal monoclonal antibody development. Human monoclonal-antibody precedents enter a pet-specific translation filter that integrates species-framework selection, target biology in the intended animal population, the feasibility of trials in client-owned animals, dose economics, veterinary-clinic workflow, and regulatory and labelling requirements. Enabling approaches—including AI-assisted design, structural screening, companion-animal tissue omics, RAG/PDF-supported evidence synthesis, and smart trial design—may reduce development bottlenecks, but their outputs require source-level verification and species-matched validation.

Because the available studies differ substantially in species, indication, molecular format, study design, comparator, endpoint, follow-up, and regulatory context, a quantitative meta-analysis was not considered appropriate. Instead, the evidence was synthesised using the translational framework shown in Figure 1.

This framework links disease biology in the intended species with species-adapted variable and Fc regions, species-matched functional assays, manufacturability, clinical trial feasibility, owner and clinic burden, animal welfare, and regulatory implementation. Emerging technologies were evaluated according to whether they address a defined veterinary clinical need rather than on technological novelty alone.

## 2. Clinical Translation of Approved and Clinical-Stage Companion-Animal Monoclonal Antibodies

### 2.1. Evidence Sources, Regulatory Verification, and Public Database Scope

Thera-SAbDab served as the entry point for identifying companion-animal antibody sequences and product names, but database inclusion alone was not treated as evidence of product status. Regulatory documents, manufacturer information, and product-specific clinical publications were used to determine which entries could be described as approved products or clinical programmes. This distinction matters because sequence availability, target rationale, and regulatory status answer different questions and carry different evidentiary weight.

Against this evidentiary background, Table 1 summarises product facts that can be traced to regulatory documents, product information, or the product-level clinical literature. Target biology, framework selection, and platform implications are developed in later sections, where they can be interpreted in context. Fc mutations, subclass assignments, and species-adaptation routes are reported only when supported by product-level, sequence-level, or primary-source evidence.

### 2.2. Anti-Cytokine mAbs for Dermatological Indications

Lokivetmab translated canine IL-31 biology into an approved dermatological therapy, yet its success does not automatically define the opportunity in feline AD [19,20,21]. The feline evidence base differs in clinically important ways: disease prevalence, pathway dominance, and phenotyping remain less well resolved in peer-reviewed studies [22]. A feline IL-31 programme should therefore be built around feline pathway evidence rather than the assumption that the canine pruritus model is directly portable.

This evidence boundary also applies to second-generation canine anti-IL-31 programmes. Target biology and publicly documented product status can be assessed from available sources, whereas claims about Fc-mediated half-life extension or dosing require sequence- and programme-level support [5,6,13]. The tables in this review therefore report Fc-engineering attributes only when product-level evidence is available.

### 2.3. Anti-NGF mAbs for Osteoarthritis Pain

NGF contributes to nociceptor sensitisation in osteoarthritis and other chronic pain states. Bedinvetmab targets canine NGF to treat OA pain in dogs, whereas frunevetmab and relfovetmab target feline NGF for OA-associated pain in cats [23,24]. Together, these products support the anti-NGF mechanism across both species, but they do not remove the need for molecule-specific validation. Binding, pharmacokinetics, safety, and Fc design must still be established independently for each species-adapted antibody. Because NGF neutralisation does not depend on ADCC or CDC, a lower-effector canine Fc can be mechanistically appropriate when product- or sequence-level evidence supports that choice [3,4,5,6].

Human anti-NGF experience provides an important safety context for these veterinary programmes. Tanezumab produced analgesic benefit in osteoarthritis but was not approved after regulatory review because of joint-safety concerns, including rapidly progressive osteoarthritis [25,26]. High target conservation strengthens translational plausibility—the mature NGF peptide is identical in dogs and cats and highly conserved relative to human NGF—but conservation cannot substitute for species-adapted backbones or species-specific safety assessment [27]. The absence of a comparable published RPOA signal in dogs and cats is reassuring but not definitive, because joint loading, owner-managed activity, case selection, and veterinary pharmacovigilance may alter how risk emerges or is detected [3,4]. Efficacy estimates also require context: owner-reported pain and mobility outcomes are clinically meaningful, yet they remain susceptible to caregiver placebo effects and study-design bias [28].

### 2.4. Oncology and Speciality mAbs

Canine lymphoma and immune-checkpoint biology support two principal oncology strategies: B-cell depletion and PD-1/PD-L1 checkpoint modulation [10,29,30,31,32,33,34,35]. Their translational appeal should not obscure the limits of the evidence. Before clinical efficacy is inferred, target expression, canine Fc-mediated effector activity, response criteria, and the precise regulatory status of each product or programme need to be linked to source-level data [5,6]. In this setting, explicit evidence boundaries are more informative than broad analogies with human oncology.

### 2.5. Infectious-Disease mAbs

CPMA illustrates a different therapeutic model: a neutralising antibody used in an acute infectious disease rather than a chronic immune-mediated condition. In a CPV-2 challenge study, early administration prevented mortality and supported product licensure [18]. This result changes how the target landscape should be organised. The CPV-2 capsid protein VP2 is no longer merely a future hypothesis; it represents an established canine antiviral-antibody target.

## 3. Canine and Feline Fc Biology as a Clinical Design Variable

### 3.1. Canine IgG Subclasses

Dogs express four IgG subclasses, designated cIgG-A, cIgG-B, cIgG-C, and cIgG-D. The aliases G1–G4 are retained only in parts of the older literature. Shared numbering should not be mistaken for functional orthology: cIgG-A, for instance, is not equivalent to human IgG1 simply because both have been called G1. Canine subclass selection must instead be grounded in canine receptor binding, effector activity, and pharmacokinetic data [11,12].

The therapeutic sequence set provides a useful, although still limited, observation. cIgG-B occurs in 10 of the 11 canine mAbs listed in Thera-SAbDab, and Bergeron et al. reported stronger engagement of canine FcγRI and FcRγIII by cIgG-B than by the other canine subclasses [5]. These findings make cIgG-B a rational starting scaffold when effector competence is required. They do not, however, establish quantitative potency, which must be measured in canine ADCC systems because cIgG-B is not a functional equivalent of human IgG1 [5,6].

Pharmacokinetic data further show why subclass labels cannot be interpreted in isolation. In the experimental setting reported by Bergeron et al., cIgG-B had an elimination half-life of 8.0 ± 1.3 days, whereas bedinvetmab showed an apparent half-life of 12–16 days in EU regulatory studies [5,36]. Molecule-specific properties, target-mediated disposition, route of administration, assay design, population, and pharmacokinetic modelling could all contribute to this difference. cIgG-A, which showed lower effector function and a longer reported half-life in the Bergeron study, may be better suited to neutralising mechanisms such as pain or cytokine blockade [5,6].

The pharmacokinetic values in Table 2 are best read as study-specific observations rather than interchangeable subclass constants. The cIgG-B estimate comes from Bergeron et al. (8.0 ± 1.3 days; *n* = 6), while the fIgG1 value derives from an intravenous frunevetmab study reported by Gruen et al. (10.1 ± 1.9 days; *n* = 8) [5,37]. Regulatory studies of individual products, including bedinvetmab, can yield different apparent half-lives because product and study context remain integral to the measurement [36].

A similar caution applies to effector-function summaries. The entries capture relative signals from species-appropriate assays and can guide candidate selection, but they are not substitutes for lot-release potency specifications. Each product still requires a mechanism-relevant potency assay in canine or feline systems.

### 3.2. Feline IgG Subclasses

Compared with canine IgG, feline IgG biology remains less systematically characterised. Strietzel et al. described abundant fIgG1 allelic forms and a less abundant putative fIgG2-like sequence, while frunevetmab provides clinical evidence that an fIgG1 therapeutic backbone can function in cats [37,38]. What the available data do not yet resolve is why frunevetmab shows its observed exposure: FcRn engagement, target-mediated disposition, reduced catabolism, and other molecule-specific factors may all contribute.

The constant-region work of Strietzel et al. provides an important foundation, identifying predominant fIgG1 allelic sequences with FcRn- and Fc-receptor-binding properties relevant to therapeutic design [38]. Even so, feline Fc biology is not yet sufficiently complete for routine product decisions. Functional characterisation of feline FcγRII/CD32 remains a particularly important gap.

### 3.3. Fc Engineering for Dosing and Clinical Feasibility

Human YTE and LS mutations demonstrate that modifying the Fc-FcRn interaction can prolong antibody exposure [39,40]. Whether the same design logic can be transferred to dogs or cats is an experimental question, not a dosing assumption. Product-level assignment of YTE- or LS-like changes requires sequence evidence, and any gain in half-life must be measured directly in the intended species. This caution is especially relevant for feline FcRn, where sequence divergence may alter the relationship between engineered binding and in vivo persistence.

## 4. Species Adaptation, Developability, and Manufacturing for Clinical Deployment

### 4.1. Classical CDR Grafting and Epitope Conservation

Once a target and Fc strategy have been selected, another question becomes decisive: can the variable region be adapted without losing binding or developability? The framework shapes CDR geometry, expression yield, aggregation risk, surface hydrophobicity, chemical liabilities, and the risk of anti-drug antibody formation. Caninization or felinization therefore involves more than grafting donor CDRs onto canine or feline VH/VL sequences; selected backmutations may be needed to preserve paratope geometry, affinity, or product behaviour. Fc selection governs effector function and half-life, whereas framework selection determines whether the binding site remains functional and manufacturable in the recipient species.

Clinical products provide useful framework precedents, although they do not establish a universal germline rule. Lokivetmab/Cytopoint, bedinvetmab/Librela, izenivetmab/Lenivia, and gilvetmab demonstrate canine or caninised frameworks in development, while frunevetmab/Solensia and relfovetmab/Portela provide feline or felinised examples. Their importance lies in showing that species-adapted variable regions can progress through the full development pathway to therapeutic products. Ranking IGHV, IGKV, or IGLV families for CMC purposes requires a different level of evidence: product sequence, species-specific germline assignment, expression titre, monomer purity, thermal stability, self-association or viscosity, formulation stability, and ADA behaviour.

Seen in this light, the marketed products are more valuable as a benchmark panel than as a league table of the ‘best germline’. They identify framework classes worth reusing or challenging experimentally: canine or caninised frameworks in chronic dermatology and pain, feline or felinised frameworks in anti-NGF pain, and canine frameworks paired with effector-competent Fc designs in oncology. VH/VL family assignments become actionable only when they are connected to expression, assembly, stability, and formulation data.

A strong framework must reconcile species identity with product performance. Germline proximity matters, but so do therapeutic precedent, predicted immunogenicity, surface-exposed framework residues and chemical liabilities, charge and hydrophobicity, VH/VL pairing, and compatibility with the donor CDRs. In practice, a useful workflow starts from marketed veterinary frameworks and combines germline assignment with liability screening, expression testing, thermal-stability analysis, self-association or viscosity measurements, and formulation stress testing. The CMC question is concrete: does the candidate express as a correctly assembled IgG, remain predominantly monomeric, avoid exposed chemical liabilities, allow concentration without excessive viscosity, and remain analytically controllable throughout cell-line development, purification, formulation, and stability studies?

A descriptive framework re-annotation undertaken for this review provides a more specific view of this problem. Variable-region sequences from Thera-SAbDab were re-annotated against IMGT-derived Canis lupus familiaris amino-acid germline references, with assignments from approved, clinical, preclinical, or patent-disclosed antibodies interpreted as therapeutic-framework precedents rather than generic repertoire statistics. These antibodies have often passed several practical filters, including expression, IgG assembly, purification, formulation feasibility, and in vivo tolerability. Their VH/VL frameworks may therefore provide stronger starting points than an untested nearest-germline scaffold with only a marginal sequence-identity advantage. In the re-annotated cohort, 14 sequence-available companion-animal antibodies had both VH and VL nearest-dog-germline assignments against a canine reference set containing 54 IGHV, 61 IGKV, and 122 IGLV amino-acid entries. Most light-chain nearest matches were IGKV (13 entries), whereas only one was assigned to IGLV. This pattern is informative for selecting a benchmark panel, but it does not establish a universal CMC preference; expression, assembly, aggregation, viscosity, purification, formulation, and stability must still be compared experimentally.

Light-chain selection should be approached with the same discipline. Feline immunoglobulin mRNA sequencing and canine κ:λ diagnostic studies demonstrate species-specific light-chain biology [41,42,43], yet neither supports a universal κ- or λ-chain advantage for therapeutic-antibody CMC. A practical programme assigns VL sequences against canine or feline IGK/IGL references and evaluates chain pairing, exposed liabilities, aggregation, expression, and formulation behaviour in the context of the complete antibody (Table S1).

### 4.2. The Feline VH Framework-Data Deficit

The feline framework gap is best understood as a deficit in engineering readiness rather than an absence of sequence data. Expressed feline IGHV genes have been reported [41], and therapeutic precedents are availableand therapeutic precedents are available [37,38]. What remains missing is a versioned, decision-ready VH/VL resource that links sequence provenance to structure, expression, stability, and therapeutic performance. Without that bridge, felinization remains dependent on a small number of mature frameworks rather than on a reusable selection system comparable to those available for human antibody engineering.

A practical programme can be organised into six stages. First, feline B-cell repertoires should be sampled across breeds, ages, sexes, tissues, geographic origins, and health or disease states, with metadata describing collection, storage, RNA quality, and sample-processing conditions. Second, paired full-length VH/VL sequences should be generated using validated single-cell or long-read workflows, preferably with molecular barcoding and explicit quality-control thresholds to reduce sequencing and chain-pairing errors. Both productive and nonproductive rearrangements should be retained where useful for germline inference, while therapeutic-framework selection should rely on productive, correctly paired sequences.

Third, sequences should be assigned against a versioned feline IGH, IGK, and IGL reference set under a shared numbering convention, with the alignment method, identity denominator, gap treatment, and assignment confidence reported. The resource should distinguish germline-supported residues from somatic substitutions and annotate framework frequency, CDR-supporting or Vernier-zone residues, VH/VL interface positions, predicted surface charge and hydrophobicity, and common chemical liabilities such as deamidation, isomerisation, oxidation, and unpaired cysteines. Structural models may be used to rank candidates, but uncertainty should be recorded and experimentally tested rather than treated as a definitive framework decision [44,45,46,47].

Fourth, a benchmark panel of recurrent and therapeutically relevant feline frameworks should be expressed as complete IgGs in a common production system. Minimum developability measurements should include expression yield, correct heavy- and light-chain assembly, monomer content by size-exclusion chromatography, thermal stability, self-association or viscosity at formulation-relevant concentrations, and stability under accelerated and freeze–thaw stress. These data should be compared using the same variable-region inserts and analytical conditions wherever possible so that framework effects are not confounded by assay or CDR differences.

Fifth, felinization should be tested through a small, rational matrix of recipient frameworks and backmutation designs rather than a single nearest-germline graft. Representative CDR grafts should be evaluated for binding kinetics, epitope retention, expression, aggregation, and stability. Backmutations should be prioritised at residues that support CDR conformation, VH/VL packing, or antigen contact, and the minimum set required to restore affinity should be distinguished from substitutions introduced only to improve developability.

Finally, lead frameworks should enter species-matched functional validation. Depending on mechanism, this should include feline antigen-binding or neutralisation assays, FcRn binding at acidic and near-neutral pH, relevant feline Fc-receptor interactions, and mechanism-appropriate potency assays. Sequence-based immunogenicity assessment may guide candidate ranking, but chronic-use programmes also require empirical anti-drug-antibody monitoring in cats. A reusable public resource should therefore connect each sequence to its metadata, assignment confidence, structural annotation, expression and stability results, formulation behaviour, and positive as well as negative grafting outcomes. This sequence-to-function structure, rather than sequence count alone, would make feline framework selection reproducible and progressively reduce the experimental burden of felinization.

### 4.3. AI-Assisted and Computational Framework Selection: Current Value and Limits

YabXnization illustrates what computational framework selection can contribute: candidate-scaffold ranking, backmutation prioritisation, and transparent comparison of species frameworks [44]. Its utility depends on the depth and quality of the underlying framework library. Canine resources are approaching the point at which specialised annotation and modelling become practical, whereas feline datasets still require greater scale, shared numbering, structural anchoring, and links to developability data. Across both species, AI-assisted adaptation is most valuable when it narrows the experimental search space; it should not replace binding, developability, immunogenicity, or CMC validation.

### 4.4. Structural Modelling and Species-Specific Validation

Structural modelling can help prioritise backmutations and anticipate stability consequences after CDR grafting, but companion-animal antibodies remain under-represented in public benchmarks [45,46,47]. The main limitation is template coverage. CDR and framework context strongly influence prediction quality; sparse canine and feline templates reduce confidence in loop-level predictions and expected RMSD values have not been adequately benchmarked for companion-animal antibodies [45,47]. Species-specific backbone geometry may also matter, although systematic canine or feline loop biases remain poorly characterised. Structural prediction should therefore be used to rank candidates for testing rather than as the sole basis for developability decisions [46].

Half-life engineering exposes the same limitation at a protein–protein interface. YTE- or LS-style extension in canine or feline Fc regions depends on species-specific Fc-FcRn interactions, yet homology models derived from human FcRn-Fc co-crystal structures may miss relevant interface divergence. Direct measurements with species-specific FcRn constructs—using surface plasmon resonance, biolayer interferometry, or comparable methods—provide the evidence needed to connect an engineered Fc sequence with a veterinary pharmacokinetic hypothesis.

A sensible workflow follows from these constraints. In silico tools can rank structural and developability risks, after which a focused panel of caninised or felinised candidates should be tested for binding, stability, and species-specific Fc behaviour before in vivo studies [45,46,47]. This sequence keeps computation close to the decisions it can support while preserving experimental validation as the final arbiter.

### 4.5. Alternative Strategies: In Vivo Immunisation and Hybridoma-Derived mAbs

Direct immunisation offers a complementary route when framework uncertainty is high. Dogs or cats can be immunised with the target antigen, and antigen-specific B-cell responses can be recovered through single-cell sequencing or hybridoma technology. The advantage is a species-adapted starting sequence. The limitations include narrower epitope coverage, lower screening throughput, and more complex production logistics than are typically available with large human display libraries.

### 4.6. Manufacturing, CMC, and Veterinary-Clinic Deployment

Approved companion-animal mAbs use CHO expression and downstream purification strategies broadly similar to those used for human biologics, but a veterinary CMC dossier cannot be treated as a scaled-down version of a human dossier. Batch sizes are smaller, price tolerance is tighter, and products are distributed through general veterinary practices, all of which increase the value of a robust and economical control strategy. Glycosylation is particularly important because Fc glycan composition can alter effector function, immunogenicity, and lot-to-lot comparability. CHO-derived glycans should not be assumed to reproduce endogenous canine or feline IgG simply because the expression platform is familiar.

The same species-specific logic applies to biological characterisation and lot-release potency. When the mechanism depends on Fc biology, binding, neutralisation, ADCC, and CDC assays should use canine or feline antigens, target cells, serum matrices, and effector-cell systems. ADA testing requires tiered screening, confirmatory, and titration assays. Cut-points should be established in drug-naive canine or feline serum. Assay sensitivity and drug tolerance should be demonstrated at clinically relevant trough concentrations. Stability programmes should also reflect use in practice by combining real-time and accelerated studies with handling conditions representative of general veterinary clinics.

## 5. Precision Target Selection for Unmet Clinical Needs

### 5.1. A Precision-Medicine Target Prioritisation Framework

How should a future companion-animal target be prioritised? The starting point is veterinary disease biology, while human antibody precedent serves as supporting context rather than as the primary justification. Table 3 focuses on future hypotheses because NGF, IL-31, PD-1, and canine parvovirus VP2 already have approved, clinical, or licensure-supporting evidence and are discussed in the product- and mechanism-focused sections.

A target becomes development-ready only when several questions converge. Does the veterinary disease share the relevant dominant biology? Is the epitope conserved, and can the proposed Fc and framework design accommodate chronic dosing, cost, and immunogenicity constraints in practice? By organising dermatology, oncology, and feline renal disease as gated hypotheses, Table 3 makes these decision points visible rather than presenting an undifferentiated wish list.

### 5.2. Dermatology Beyond IL-31: Endotype-Guided Development

Lokivetmab establishes canine atopic dermatitis as a clinical antibody indication, but it does not make the human dermatology pipeline portable. IL-4Rα and IL-17A programmes are credible only when canine or feline cytokine biology, infection risk, and clinical phenotype support them; earlier canine cytokine-cloning studies underscore the need for species-specific reagents and assays [5,6,49]. IL-17A blockade may be effective in selected human inflammatory skin diseases, yet the veterinary context includes barrier inflammation, pyoderma, and antimicrobial host defence. Those features place IL-17A in an exploratory category with a clear infection-risk boundary rather than making it a near-term analogue of human psoriasis therapy [50,51].

The comparison between canine anti-IL-31 therapy and human anti-IL-4Rα therapy reveals why phenotype alone is insufficient. Similar-appearing inflammatory skin diseases may be driven by different dominant cytokines. Canine IL-31 biology and lokivetmab efficacy make IL-31 a central target in canine pruritus, whereas its role in feline disease remains less certain [21,52]. Sequence divergence adds another layer: canine IL-31 shares limited amino-acid identity with human IL-31, helping to explain why the human and canine programmes required separate antibodies [21]. Until feline cross-reactivity and pathway dominance are demonstrated in peer-reviewed studies, canine conclusions should not be extended to cats. The broader lesson also applies in reverse. Strong human relevance, as seen with PCSK9 in cardiovascular disease, does not create a veterinary indication without a defined species, lipid phenotype, and clinical need.

### 5.3. Oncology: Biomarker-Guided Checkpoint Inhibition and B-Cell Depletion

Companion-animal oncology offers attractive translational opportunities, although the evidence remains uneven across species, targets, and tumour types. Feline comparative-oncology evidence and species-specific studies of the canine PD-1/PD-L1 axis support selected programmes; however, the human antibody pipeline should be used primarily to generate testable hypotheses rather than to provide a ready-made veterinary roadmap [29,33,35,53,54]. Gilvetmab illustrates this point. It should be interpreted as a species-specific PD-1 programme whose indication, licence status, and endpoint data are defined by product-level sources rather than generalised across veterinary tumours.

CD20 remains compelling in canine B-cell lymphoma because anti-CD20 therapy transformed the treatment of human B-cell malignancies, but veterinary development begins where that analogy ends. A canine programme requires confirmation of tumour CD20 expression and heterogeneity, species-matched assessment of Fc-mediated effector function, and pharmacodynamic monitoring of B-cell depletion before clinical efficacy can be meaningfully evaluated [29,30,32]. Human precedent supports candidate nomination; it cannot substitute for canine clinical evidence.

HER2/ERBB2 offers a different kind of future opportunity. No veterinary HER2 product is marketed, yet human therapies establish targetability, and a defucosylated mouse-dog chimeric anti-HER2 antibody has shown binding, canine effector-function activity, and antitumour effects in HER2-positive canine xenograft models [48]. Moving from that proof of concept to a veterinary programme would require tumour-specific HER2 scoring, confirmation of epitope recognition, a defined internalisation or effector-function rationale, and evidence from spontaneous HER2-high canine or feline tumours.

### 5.4. Feline Renal Disease: Biomarker-Gated Opportunities

Feline CKD, often characterised by chronic tubulointerstitial nephritis and progressive renal dysfunction, represents an area of high unmet need for future target discovery [55]. Yet the public product landscape contains no established feline renal or nephritis antibody programme. This absence sets the evidentiary boundary: renal concepts should remain disease-area hypotheses until feline target expression, patient stratification, a credible therapeutic window, and product-level support are available.

What would make a feline renal programme credible? Target expression is only the entry point. IRIS-stage-linked samples, stratification by proteinuria and blood pressure, longitudinal biomarkers, confirmation of expression in feline renal tissue, and a therapeutic window that separates disease modification from disruption of compensatory physiology would all be required. Feline nephritis and CKD are therefore high-need opportunities, but they remain exploratory in Table 3.

### 5.5. Pain Hypotheses Beyond NGF

CGRP shows how a validated human pain target can still lack a clear veterinary entry point. Anti-CGRP mAbs are established migraine preventives in humans, but companion-animal development would require canine and feline receptor pharmacology, epitope-conservation analysis, and a pain model capable of distinguishing CGRP-driven from NGF-driven nociception. Without that disease-specific bridge, human success provides rationale rather than readiness.

## 6. Next-Generation Modalities and Technology-Enabled Delivery

### 6.1. Start from the Veterinary Clinical Use Case, Not the Modality

The approved companion-animal mAbs—lokivetmab, bedinvetmab, frunevetmab, relfovetmab, izenivetmab, tirnovetmab, and CPMA—share a practical pattern: a species-adapted IgG administered at intervals ranging from monthly to quarterly can be sufficient when target biology and clinic workflow are aligned. This observation provides the baseline against which newer formats should be judged. Bispecific antibodies, ADCs, VHHs, mRNA-encoded antibodies, and checkpoint combinations are unlikely to replace conventional IgG broadly; their value lies in indications that a well-characterised species-adapted IgG cannot adequately address.

### 6.2. Oncology: Rationale for Format Escalation and Checkpoint Combination

When is format escalation justified? Canine oncology provides the clearest answer because some tumours may require Fc-mediated effector function, dual-target engagement, or tissue penetration beyond what a conventional species-adapted IgG can deliver. The four programmes below are considered in that use-case-driven context rather than as a catalogue of platform novelty.

Combined PD-1 and CTLA-4 blockade is one of the more immediately testable concepts in canine melanoma and MCT. Gilvetmab supports PD-1 as a canine target, while a caninised anti-CTLA-4 antibody has entered first-in-dog evaluation [11]. Human experience with ipilimumab plus nivolumab suggests that dual checkpoint blockade can deepen responses in selected tumour types, although the magnitude of benefit and safety profile cannot be assumed in dogs. A caninised bispecific could consolidate treatment into one injection per visit and potentially support cooperative FcγR engagement for Treg depletion; cIgG-B is a plausible starting subclass when ADCC-mediated depletion is part of the intended mechanism.

Anti-RANKL therapy in canine osteosarcoma has a similarly coherent translational rationale. Osteosarcoma is the most common primary bone tumour in large-breed dogs and serves as a spontaneous model of paediatric human disease. A caninised antibody analogous to denosumab could reduce osteolysis, preserve weight-bearing function, and potentially delay the need for amputation. Species-specific engineering would still be required because canine RANKL may diverge at relevant epitopes, but the clinical problem and comparative-oncology value justify further investigation.

Feline mammary carcinoma raises a different question: could an ADC provide activity where unconjugated IgG is insufficient? A subset of feline mammary carcinomas overexpresses HER2, and these spontaneous tumours can be biologically aggressive. A felinised anti-HER2 IgG carrying a cleavable auristatin or maytansine payload could translate the design logic of HER2-directed ADCs into this setting [48,56]. Before such a programme is credible, feline HER2 IHC/FISH scoring must be validated and the felinised Fc characterised.

CD20-targeting bispecifics in canine B-cell lymphoma extend the same use-case logic. Single-agent anti-CD20 depletion relies on ADCC and CDC; adding a T-cell- or NK-cell-engaging arm, such as anti-CD3 or anti-CD16, could increase the depth of response. Format selection should occur only after canine CD20 expression and heterogeneity, effector-cell activity, and pharmacodynamic B-cell-depletion endpoints have been defined [30,32].

### 6.3. Infectious Disease and Outbreak-Responsive Antibody Platforms

Conditional approval of CPMA opens a therapeutic category centred on acute infectious disease rather than chronic management. Anti-feline coronavirus (FCoV) mAbs are a plausible extension because untreated FIP is usually fatal, while current small-molecule regimens such as GS-441524 and GC376 require prolonged daily dosing and may have uneven regulatory availability. A neutralising mAb against a conserved receptor-binding region of the feline coronavirus spike protein could, in principle, support short-course treatment or prophylaxis in high-risk shelter populations, following the mechanistic precedent of CPMA against CPV-2. Available feline germline resources make felinization technically conceivable, although target conservation and in vivo neutralisation would need to be demonstrated.

The same development pathway may be relevant beyond FIP. CPMA suggests that the USDA conditional-licence framework could support outbreak-response mAbs for canine distemper virus, feline calicivirus, or shelter emergencies in populations with uncertain vaccination histories. Acute-use products may tolerate immunogenicity and pharmacokinetic profiles that differ from those required for chronic therapies, provided that the neutralising window and required treatment duration are clearly defined.

### 6.4. VHH, Multispecific, and mRNA Formats: Delivery Opportunities and Species-Specific Constraints

VHH domains are attractive for local or compartment-specific delivery, but their favourable physicochemical properties do not eliminate the need to optimise molecular configuration, tissue exposure, and species-specific pharmacokinetics, including renal clearance and albumin binding [57,58,59,60]. In canine ocular disease, intravitreally administered VHHs may reach posterior-segment targets at lower injection volumes and with greater tissue penetration than full-length IgG. Systemic exposure might be extended with Fc–VHH fusions or albumin-binding VHHs directed against canine rather than human serum albumin. The key parameter, however, is empirical: the canine VHH–albumin dissociation constant must be measured directly rather than inferred from rodent studies.

Tandem VHH constructs may also address problems of target geometry. An anti-IL-31 × anti-IL-4 molecule for refractory canine atopic dermatitis, for example, could engage two soluble cytokines without some of the steric constraints encountered by full-length bispecific IgGs [61]. Before pharmacokinetic studies are justified, the affinity, epitope, and simultaneous-binding properties of the canine IL-4 and IL-31 arms need to be established in the same molecular context.

Outbreak management provides a specific, although still hypothetical, niche for mRNA-encoded antibodies. A single dose of mRNA encoding a felinised anti-FCoV IgG, delivered in a lipid nanoparticle optimised for feline tolerability, could in principle generate circulating antibody within a clinically useful timeframe and offer a deployable option during shelter outbreaks. The concept remains speculative because feline LNP biodistribution, translation kinetics, and immunogenicity have not yet been defined. Those data, rather than platform precedent in other species, would determine whether the approach is viable.

### 6.5. AI-Assisted Design and Decision Support

The near-term role of AI in companion-animal antibody development is best viewed as decision support rather than autonomous discovery. Current applications with a defensible technical basis include germline assignment, framework ranking, backmutation prioritisation, structure-based triage, and screening for sequence- or structure-associated developability liabilities [44,46,47,62]. These tools may help prioritise a smaller set of candidates for experimental testing, but their reliability is constrained by the limited size and uneven annotation of canine and feline sequence datasets, inconsistent numbering and metadata, sparse species-specific structural templates, and the absence of broadly validated veterinary benchmarks for CDR-H3, FcRn, and Fc-receptor interfaces. Predictions should therefore be reported with their model, input data, confidence measures, and decision rule, and should not be treated as evidence of binding, pharmacokinetics, safety, or manufacturability.

The economic value of computational triage is similarly conditional. Companion-animal biologics are developed for comparatively small and often owner-funded markets, in which extensive construct panels and repeated assay cycles can rapidly erode commercial feasibility. AI-assisted ranking is useful only when it reduces the number of molecules that must be expressed and tested or shortens an experimentally validated design cycle. It cannot replace species-matched expression, binding, FcRn and Fc-receptor measurements, mechanism-relevant potency assays, stability and aggregation testing, immunogenicity assessment, or CMC development. A realistic workflow therefore uses computation to prioritise experiments within a closed sequence-to-function loop, with advancement decisions based on reproducible wet-laboratory and in vivo evidence rather than on the output of a single model.

For felinised or caninised programmes, the most practical implementation is a transparent ranking workflow in which experimentally observed VH/VL sequences define the candidate space, computational methods prioritise frameworks and backmutations, and a limited panel is tested under common analytical conditions. The value of the approach should be judged by measurable reductions in construct number, assay burden, time, and development cost while maintaining or improving binding, developability, and species-specific function. More ambitious applications, including de novo target discovery or autonomous antibody design from predominantly human training data, remain research hypotheses until companion-animal datasets and prospective validation are substantially improved.

### 6.6. A Practical Translational Deployment Hierarchy

The products and preclinical evidence reviewed here support a use-case-driven hierarchy for format selection. Species-adapted IgG remains the default for chronic pain, dermatitis, and neutralising antiviral indications when clinic-compatible dosing and manufacturing precedents already exist. Fc-engineered IgG becomes relevant when canine or feline effector function—ADCC, CDC, or Treg depletion—is required and demonstrable in species-appropriate assays. More complex modalities, including checkpoint combinations, ADCs, bispecifics, VHHs, mRNA delivery, and gene-encoded antibodies, should be reserved for situations in which conventional IgG cannot adequately address target biology, tissue-exposure limitations, outbreak logistics, or manufacturing timelines. Regardless of format, several requirements should be met before pivotal or GLP-equivalent studies. These include confirmed target biology in the intended species, Fc function in companion-animal effector cells, and potency assays in species-matched systems. Candidates should also show acceptable framework developability, ADA-assay readiness, and feasible handling in veterinary clinics.

## 7. Conclusions: A Clinical and Translational Research Agenda

Monoclonal antibodies are now established in companion-animal pain, dermatology, and selected infectious diseases, whereas oncology programmes are advancing under more indication- and endpoint-specific evidence standards [8,9,10,12,14,15,16,17,18]. The central conclusion of this review is that progress will depend less on importing additional human formats than on strengthening the veterinary evidence infrastructure. Three priorities are closely linked. First, public feline framework resources should connect sequence data with structure and developability [41]. Second, canine and feline FcRn and Fc-receptor biology require decision-relevant validation [5,6,38]. Third, CMC and regulatory expectations should reflect the economics and clinical realities of small markets for veterinary biologics.

Our sequence review suggests a practical route from these priorities to implementation. Diverse feline B-cell repertoires should be sequenced and curated under shared numbering and germline conventions. Leading VH/VL frameworks can then be benchmarked for expression, stability, and formulation behaviour, while Fc engineering is evaluated in feline FcRn and Fc-receptor assays before in vivo studies. The goal is a sequence-to-function resource that makes felinization reproducible and evidence-based rather than a case-by-case exercise.

## Figures and Tables

**Table 1:** Canine and feline therapeutic mAbs with public sequence data in the Thera-SAbDab review snapshot [7].

Target	Species	Indication	Approved Product(s) (Brand)	Regulatory Status	Ref.
IL-31	Canine	Atopic/allergic dermatitis	Lokivetmab (Cytopoint)	USDA and EMA approval	[12,13]
IL-31	Canine	Atopic/allergic dermatitis	tirnovetmab (Befrena)	USDA approval (December 2025)	[12,13]
NGF	Canine	OA pain	Bedinvetmab (Librela)	FDA and EMA approval	[8,14,15]
NGF	Canine	OA pain	izenivetmab (Lenivia)	EMA approval (November 2025)	[8,14,15]
NGF	Feline	OA pain	Frunevetmab (Solensia)	FDA and EMA approval	[9,16,17]
NGF	Feline	OA pain	Relfovetmab (Portela)	EMA and HC approval (2025)	[9,16,17]
PD-1	Canine	Canine oral melanoma	Gilvetmab	USDA conditional licence; endpoint data require interpretation from public sources	[10]
CPV-2	Canine	Canine parvovirus	CPMA	USDA conditional licence (2023)	[18]

Notes: mAb, monoclonal antibody; OA, osteoarthritis; USDA, United States Department of Agriculture; EMA, European Medicines Agency; FDA, United States Food and Drug Administration; HC, Health Canada; CPV-2, Canine parvovirus type 2.

**Table 2:** Canine and feline IgG subclass properties and therapeutic usage.

Species	Subclass	FcγR Binding Profile	ADCC Activity	CDC Activity	Intrinsic Subclass PK Property	Reported Experimental PK Observation
Canine	cIgG-A	FcγRI-predominant; weak FcγRIII binding	Low	Low	No consistent constant-region-defined PK profile; half-life varies by variable region	—
Canine	cIgG-B	Binds both FcγRI and FcγRIII (CD16)	High	Moderate	Extended serum persistence typical of effector-competent IgG subclasses	8.0 ± 1.3 d (*n* = 6; experimental canine IgG construct)
Canine	cIgG-C	Minimal FcγR engagement; low effector function	Low	Low	No consistent constant-region-defined PK profile; half-life varies by variable region	—
Canine	cIgG-D	Immunomodulatory phenotype; FcγRIIb-biassedbiased binding	Low	Low	No consistent constant-region-defined PK profile; half-life varies by variable region	—
Feline	fIgG1	Binds both FcγRI and FcγRIII	Moderate to high	Moderate	Extended serum persistence typical of effector-competent IgG subclasses	10.1 ± 1.9 d (*n* = 8; intravenous frunevetmab)
Feline	fIgG2	Weak FcγR engagement; minimal effector function	Low	Low	Insufficient product-level data to define consensus subclass PK profile	—

Notes: ADCC, antibody-dependent cellular cytotoxicity; CDC, complement-dependent cytotoxicity; PK, pharmacokinetics; FcγR, Fc gamma receptor; d, days; —, no study-specific value available. Qualitative classifications (High/Moderate/Low) are assigned based on relative in vitro binding affinity and effector function magnitude across peer-reviewed published studies of canine and feline IgG subclasses: “High” indicates robust, reproducible receptor binding and effector activity; “Moderate” indicates intermediate functional activity; “Low” indicates minimal or undetectable binding/effector function. Intrinsic subclass PK properties represent consensus characteristics of the IgG constant region; experimental PK observations are study-specific values from individual antibody molecules and are not generalizable to all antibodies of the same subclass.

**Table 3:** Future companion-animal antibody target hypotheses after excluding established product axes.

Candidate Target	Veterinary Rationale	Key Go/No-Go Evidence
IL-4Rα/type 2 axis	Refractory canine or feline AD with a type 2-high endotype dominated by IL-4/IL-13, beyond control of IL-31-mediated pruritus; plausible only in a confirmed type 2-predominant subgroup.	Advance only if skin transcriptomics and cytokine profiling confirm IL-4/IL-13 dominance in steroid-refractory AD; do not extrapolate from human dupilumab data alone.
IL-17A/IL-23 axis	Barrier inflammation with neutrophilic or microbial features; biologically plausible, but infection risk from suppression of host defence is the critical go/no-go consideration.	Define the disease subgroup and microbiological context and establish an acceptable infection-risk boundary before antibody nomination.
CD20/B-cell depletion	Canine B-cell lymphoma; a coherent depletion target for which Fc-mediated ADCC and CDC may contribute to clinical response.	Confirm tumour CD20 expression and heterogeneity, evaluate canine Fc-mediated effector function and pharmacodynamic B-cell depletion, and relate these measures to clinical endpoints.
HER2/ERBB2	HER2-positive feline mammary and canine epithelial tumours; a defucosylated anti-HER2 antibody provides proof of concept for Fc-enhanced cytotoxicity in vitro [48].	Standardise feline HER2 IHC/FISH scoring, confirm internalisation and Fc function, and advance only in spontaneous HER2-high tumours.
Feline CKD/nephritis	High unmet need in feline renal disease; inflammatory and fibrotic pathways remain exploratory in the absence of confirmed target-expression data.	Require feline renal target expression, linkage to IRIS stage, proteinuria and blood pressure endpoints, and a chronic safety window before nomination.
CTLA-4/PD-1 combination	Canine MCT and melanoma; gilvetmab supports PD-1 biology, and a caninised anti-CTLA-4 antibody has entered first-in-dog evaluation; dual blockade may deepen responses relative to monotherapy [11].	Confirm cIgG-B suitability for ADCC-mediated Treg depletion, measure CD4+FOXP3+ depletion as a pharmacodynamic endpoint, and pilot dual-agent safety in stage III–IV MCT or melanoma before selecting a bispecific format.

Notes: AD, atopic dermatitis; ADCC, antibody-dependent cellular cytotoxicity; CDC, complement-dependent cytotoxicity; CD20, cluster of differentiation 20; CKD, chronic kidney disease; CTLA-4, cytotoxic T-lymphocyte-associated protein 4; ERBB2/HER2, erb-b2 receptor tyrosine kinase 2/human epidermal growth factor receptor 2; Fc, fragment crystallisable; FISH, fluorescence in situ hybridisation; FOXP3, forkhead box P3; IHC, immunohistochemistry; IL, interleukin; IL-4Rα, interleukin-4 receptor alpha; IRIS, International Renal Interest Society; MCT, mast cell tumour; PD-1, programmed cell death protein 1; Treg, regulatory T cell.

## Data Availability

No new data were created or analyzed in this study.

## Supplementary Materials

The following supporting information can be downloaded at: https://www.mdpi.com/article/10.3390/vetsci13080778/s1, Table S1: Re-annotation of 14 publicly available companion-animal antibodies against canine germline V genes.

## Abbreviations

The following abbreviations are used in this manuscript:

ADA	anti-drug antibody
AD	atopic dermatitis
ADCC	antibody-dependent cellular cytotoxicity
ADC	antibody-drug conjugate
CDC	complement-dependent cytotoxicity
CDR	complementarity-determining region
CGRP	calcitonin gene-related peptide
cIgG-A, cIgG-B, cIgG-C, cIgG-D	canine IgG subclasses A–D (G1–G4 aliases in parts of the literature)
CKD	chronic kidney disease
CMC	chemistry, manufacturing, and controls
Fc	fragment crystallisable (constant region of IgG)
FcγRIII	Fc gamma receptor III (CD16)
FcRn	neonatal Fc receptor
fIgG1, fIgG2	feline IgG subclasses 1 and 2 [38]
IMGT	International ImMunoGeneTics information system
LNP	lipid nanoparticle
LS	M428L/N434S Fc mutations for half-life extension (human IgG1)
mAb	monoclonal antibody
NGF	nerve growth factor
NSAID	non-steroidal anti-inflammatory drug
OA	osteoarthritis
Thera-SAbDab	Therapeutic Structural Antibody Database [7]
VHH	variable domain of heavy-chain-only antibody (nanobody)
YTE	M252Y/S254T/T256E Fc mutations for half-life extension (human IgG1)
