# Supplementary material for: Therapeutic Monoclonal Antibodies as Advanced Therapies for Companion Animals: Species Adaptation, Fc Biology, Clinical Translation, and Future Platforms
Source: Vet Sci. 2026 Aug 3;13(8):778. doi: 10.3390/vetsci13080778 (PMC13517563; doi:10.3390/vetsci13080778)
Supplement: Supplementary file 1 [file vetsci-13-00778-s001.zip › vetsci-4478535-supplementary.pdf]

Supplementary Table S1. Re-annotation of 14 publicly available companion-animal antibodies against canine germline V genes

Antibody	Species	Nearest canine IGHV	VH V-segment identity (%)	VH FR identity (%)	Nearest canine IGKV/IGLV	VL V-segment identity (%)	VL FR identity (%)
Anivovetmab	Canine	IGHV4-1*01	58.9	67.6	IGKV IGKV3-18*01	67.0	71.4
Bedinvetmab	Canine	IGHV3-19*01	91.8	94.4	IGLV IGLV1-141*01	88.8	91.3
Blontuvetmab	Canine	IGHV1-30*01	60.2	62.0	IGKV IGKV4-15*01	69.1	65.7
Cirevetmab	Canine	IGHV1-30*01	88.8	100.0	IGKV IGKV3-18*02	85.2	95.7
Dovanvetmab†	Feline	IGHV1-30*01	65.3	76.1	IGKV IGKV3-18*01	72.7	80.0
Frunevetmab†	Feline	IGHV3-9*01	59.8	71.8	IGKV IGKV2-16*01	68.2	78.6
Gilvetmab	Canine	IGHV3-54*01	70.4	83.1	IGKV IGKV2-10*03	84.1	94.3
Izenivetmab	Canine	IGHV3-67*01	82.7	97.2	IGKV IGKV3-18*02	87.5	100.0
Lokivetmab	Canine	IGHV3-67*01	87.8	97.2	IGKV IGKV3-18*02	85.2	100.0
Nolavetbart	Canine	IGHV4-1*01	81.1	90.1	IGKV IGKV4-15*01	79.5	88.6
Ranevetmab	Canine	IGHV3-2*01	72.2	87.3	IGKV IGKV3-18*02	83.0	94.3
Relfovetmab†	Feline	IGHV3-67*01	76.5	90.1	IGKV IGKV3-18*01	73.9	82.9
Tamtuvetmab	Canine	IGHV3-31*01	66.3	71.8	IGKV IGKV3-18*01	67.0	72.9
Tirnovetmab	Canine	IGHV4-1*01	61.1	66.2	IGKV IGKV3-18*01	63.6	71.4

Legend: V-segment identity denotes amino-acid identity to the nearest canine IMGT V allele across shared Kabat-numbered positions preceding CDR3, including CDR1 and CDR2. FR identity was calculated over FR1–FR3 only. CDR3 and FR4 were excluded. †Feline antibodies were evaluated by cross-species comparison with canine germlines. Public VH/VL sequences were obtained from Thera-SAbDab; canine V-gene references were obtained from IMGT. Two author-designed sequences were excluded.
